# Quantifying EHR and Policy Factors Associated with the Gender Productivity Gap in Ambulatory, General Internal Medicine

**DOI:** 10.1007/s11606-023-08428-5

**Published:** 2023-10-16

**Authors:** Huan Li, Lisa Rotenstein, Molly M. Jeffery, Hyung Paek, Bidisha Nath, Brian L. Williams, Robert M. McLean, Richard Goldstein, Teryl K. Nuckols, Lalima Hoq, Edward R. Melnick

**Affiliations:** 1 Department of Emergency Medicine, Yale University School of Medicine, New Haven, CT USA; 2 Computational Biology and Bioinformatics, Yale School of Medicine, New Haven, CT USA; 3 Department of Medicine, Brigham and Women’s Hospital, Boston, MA USA; 4 Harvard Medical School, Boston, MA USA; 5 Department of Emergency Medicine, Mayo Clinic, Rochester, MN USA; 6 Division of Health Care Delivery Research, Mayo Clinic, Rochester, MN USA; 7 Information Technology Services, Yale New Haven Health System, New Haven, CT USA; 8 Northeast Medical Group, Stratford, CT USA; 9 Department of Internal Medicine, Yale School of Medicine, New Haven, CT USA; 10 Division of General Internal Medicine, Cedars-Sinai Medical Center, Los Angeles, CA USA; 11 Cedars-Sinai Medical Center, Los Angeles, CA USA; 12 Department of Biostatistics (Health Informatics), Yale School of Public Health, New Haven, CT USA

## Abstract

**Background:**

The gender gap in physician compensation has persisted for decades. Little is known about how differences in use of the electronic health record (EHR) may contribute.

**Objective:**

To characterize how time on clinical activities, time on the EHR, and clinical productivity vary by physician gender and to identify factors associated with physician productivity.

**Design, Setting, and Participants:**

This longitudinal study included general internal medicine physicians employed by a large ambulatory practice network in the Northeastern United States from August 2018 to June 2021.

**Main Measures:**

Monthly data on physician work relative value units (wRVUs), physician and practice characteristics, metrics of EHR use and note content, and temporal trend variables.

**Key Results:**

The analysis included 3227 physician-months of data for 108 physicians (44% women). Compared with men physicians, women physicians generated 23.8% fewer wRVUs per month, completed 22.1% fewer visits per month, spent 4.0 more minutes/visit and 8.72 more minutes on the EHR per hour worked (all *p* < 0.001), and typed or dictated 36.4% more note characters per note (*p* = 0.006). With multivariable adjustment for physician age, practice characteristics, EHR use, and temporal trends, physician gender was no longer associated with productivity (men 4.20 vs. women 3.88 wRVUs/hour, *p* = 0.31). Typing/dictating fewer characters per note, relying on greater teamwork to manage orders, and spending less time on documentation were associated with higher wRVUs/hour. The 2021 E/M code change was associated with higher wRVUs/hour for all physicians: 10% higher for men physicians and 18% higher for women physicians (*p* < 0.001 and *p* = 0.009, respectively).

**Conclusions:**

Increased team support, briefer documentation, and the 2021 E/M code change were associated with higher physician productivity. The E/M code change may have preferentially benefited women physicians by incentivizing time-intensive activities such as medical decision-making, preventive care discussion, and patient counseling that women physicians have historically spent more time performing.

## INTRODUCTION

The physician gender pay gap has continued for decades with compensation for women physicians persistently 25% lower than that of men physicians even after adjusting for many factors that may be associated with physician compensation, including age, hours worked, years of experience, specialty, time period, location, setting, work-life balance preferences, and leadership position.^1–6^ How physicians spend their time is a fundamental factor that drives compensation. In a cross-sectional analysis of 24.4 million primary care office visits in 2017, women physicians generated equal revenue per visit but spent 15.7% more time with each patient, thereby generating 10.9% less revenue across 10.8% fewer visits than their male counterparts.^7^ That study concluded that lower visit volume and more time on direct patient care per visit drive the gender pay gap for primary care physicians (PCPs).

There are multiple potential contributors to the additional time women physicians spend per visit including differential patient expectations and evidence that women physicians spend more time with patients listening, providing preventive care, and counseling^8^ which is reflected in a higher volume of EHR inbox messages.^9^ Additionally, although approximately half of physician work time is spent using the EHR^10–12^ regardless of gender, EHR work burden disproportionately affects women physicians who spend more time on the EHR than men physicians, specifically on documentation, inbox management, and EHR work outside of scheduled clinical hours.^9, 13–16^

Quantifying EHR use and other factors that contribute to women physicians completing fewer visits per hour and thus generating fewer relative value units (RVUs; a measure of clinical productivity) could identify more targeted solutions to the gender pay gap. For example, in January 2021, the American Medical Association and Centers for Medicare and Medicaid Services issued changes to coding regulations for evaluation and management (E/M) visits meant to streamline the documentation needed to apply certain codes and the allowance of time-based billing that compensates physicians for previously non-billable time-based activities such as patient counseling.^6, 17^ Although on a national level and in the period immediately after implementation, these changes were not found to significantly affect EHR time,^17^ their specific effect for internal medicine and women physicians is unclear.

To identify factors associated with physician productivity—and how they may differ by physician gender—we paired EHR data with locally available data regarding PCP and practice characteristics, EHR use, and clinical productivity. Using this data, we aimed to characterize: (1) how measures of clinical activity and EHR time differ for men and women physicians, (2) how clinical productivity (measured in work RVUs, subsequently referred to as *wRVUs*) differs by physician gender, and (3) the determinants of productivity (measured in wRVUs) across ambulatory internal medicine physicians and whether differences in productivity by gender persist in adjusted analyses.

## METHODS

### Study Design, Setting, and Participants

This longitudinal study examined physician productivity, EHR use, and physician and practice characteristics for all ambulatory, general internal medicine physicians employed by a large ambulatory, non-teaching, community practice network in the Northeast region of the USA from August 2018 to June 2021. The practice network operates on a single installation of the Epic EHR (Epic Systems) and uses a productivity-based compensation model. The study protocol was approved by the practice network’s institutional review board (protocol #072105) with a waiver of informed consent since no protected health information was collected and all data were de-identified. Reporting follows the Strengthening the Reporting of Observational Studies in Epidemiology (STROBE) guidelines. Based on results of a previous feasibility analysis to ensure only inclusion of ambulatory EHR use data, physician-months with fewer than 30 scheduled clinical hours were excluded.^12, 18^ One male physician with missing data was excluded from the multivariable analysis.

### Data Sources and Variables

Monthly data were obtained from three sources: RVU and physician demographic data from the practice network’s operational database and human resources rosters, scheduling data from the Epic Clarity database, and EHR use data from the Epic Signal platform.

The primary dependent variable was productivity per completed hour of clinical work, as reflected by work relative value units per hour (wRVU/hour). Used to determine physician compensation for services rendered, the wRVU has become the de facto standard measure for physician productivity in the US fee-for-service healthcare delivery system.^19^ Non-work RVU components were not included. wRVUs are reported by month, by hour worked (aggregated wRVUs per month divided by completed clinical hours per month), and by visit (wRVUs per month divided by the number of appointments per month). wRVUs were normalized per hour of completed clinical time to (1) facilitate a common denominator, (2) since women are more likely to practice part-time, and (3) to account for time physicians spend in other activities (such as administration, teaching, or research).

Physician gender was the primary explanatory variable. Other independent variables encompassed four major categories: physician characteristics, practice characteristics, EHR use measurements, and temporal trends that may have affected physician productivity.

Physician characteristics variables included physician age category (25–44, 45–54, 55–64, and ≥ 65 and older) and gender. Practice characteristic measures for each physician included panel count (unique patients seen in the prior 2 years for whom the physician was listed as the patient’s Primary Care Physician in the EHR), and panel complexity (average Epic General Adult risk score of the physician’s panel), panel gender ratio (proportion of female patients in an individual physician’s panel for the study period), visits per month, completed clinical hours per month (number of clinical hours actually worked), minutes per visit ((completed clinical hours per month/visits per month) × 60), and proportion of available appointments filled.

EHR use measurements included time-based core EHR use metrics normalized to 1 h of completed clinical time (total, work outside of scheduled clinical hours, documentation, inbox, orders, chart review) team support (the proportion of orders with team contribution), and documentation characteristics.^20^ Note composition variables included the proportion of note characters typed or dictated by the physician (vs. by a scribe, from templates, copy paste, etc.) and the proportion of note characters contributed by physician vs. other team members.

Temporal trend variables included the COVID-19 wave and changes to coding regulations for the evaluation and management (E/M). Given major disruptions in care delivery and productivity due to the pandemic,^21^ a COVID wave variable was created (wave 0: February 2020 and earlier, wave 1: March–June 2020, wave 2: July–October 2020, wave 3: November 2020–March 2021, wave 4: April–June 2021). The E/M code change variable was defined as before or after January 2021.^17, 22^

### Statistical Analysis

Data for all physicians, and for women and men physicians, were summarized separately with descriptive statistics aggregated at the individual physician level by month. We then performed bivariate comparisons of physician characteristics, practice characteristics, EHR use measures, and wRVUs with chi-square for categorical variables and the Kruskal–Wallis test for continuous variables. Statistical significance was set at *p* < 0.05, and all tests were 2-tailed.

Associations between physician productivity, gender, and EHR use patterns were examined monthly for each physician using generalized estimating equations (GEEs) for panel data (xtgee in Stata using Poisson models with a log link and exchangeable correlation structure) to account for within-physician correlation due to the multiple observations for each physician. The dependent variable for all models was wRVUs with the natural log of completed clinical hours in the month included in the regression as an exposure variable (i.e., with the coefficient constrained to 1) to standardize the model to estimate wRVUs per completed clinical hour.

After finding that the E/M code change was associated with wRVUs/hour, we performed a random effects Poisson regression analysis to explore the association between physician gender and wRVUs/hour after the E/M code change. Physician gender, a binary variable for pre-/post-E/M code change, and the interaction of the two were included in the model. Huber-White standard errors were specified for all models. Incident rate ratios (IRRs) were selected for reporting model results for ease of interpretation; for example, an IRR of 1.5 indicates a 50% increase in the outcome variable. Predictive margins were calculated to examine gender differences in wRVUs. All statistical analyses were conducted in Stata/BE version 17.0 (StataCorp).

## RESULTS

### Participants and Descriptive Data

A total of 108 unique internal medicine physicians met inclusion criteria, representing 3227 physician-months of data. Of the physicians, 48 (44.4%) were women, and 32 (29.6%) were aged 45 to 54 (Table 1). In general, women physicians were younger. At the practice level, women physicians’ panels had similar complexity but had 359 fewer patients (26.8% smaller) with a 29 percentage points more women patients (75% female patients vs. 46%, *p* < 0.001).

**Table 1:** Demographic Characteristics, Productivity, EHR Use, and Note Content by Physician Gender

Variable	All (*N* = 108)	Men (*N* = 60)	Women (*N* = 48)	Difference (%)	*p*-value
	Mean (SD)	Mean (SD)			
RVUs					
Total work RVUs	375.43 (141.0)	419.91 (144.4)	319.83 (115.7)	− 100.08 (23.8)	< 0.001
wRVUs/hour	4.06 (1.1)	4.42 (1.2)	3.61 (0.8)	− 0.81 (18)	< 0.001
wRVUs/visit	1.83 (0.3)	1.86 (0.4)	1.79 (0.2)	− 0.07 (5.2)	0.46
Characteristic	Physician, no. (%)	Physician, no. (%)			
Physician gender	N/A	N/A	N/A	N/A	N/A
Age, years					
25–44	29 (26.9)	12 (20)	17 (35.4)	5 (41.7)	0.44^*^
45–54	32 (29.6)	17 (28.3)	15 (31.3)	− 2 (11.8)	
55–64	28 (25.9)	15 (25)	13 (27.1)	− 2 (13.3)	
65 +	19 (17.6)	16 (26.7)	3 (6.3)	− 13 (81.3)	
	Mean (SD)	Mean (SD)			
Practice characteristics					
Panel count	1179.7 (564.2)	1339.2 (611.1)	980.3 (417.5)	− 358.9 (26.8)	< 0.001
Panel complexity	1.57 (0.41)	1.63 (0.45)	1.50 (0.35)	− 0.13 (8.0)	0.10
Panel gender ratio, proportion female	0.58 (0.16)	0.46 (0.07)	0.75 (0.07)	0.29 (63.0)	< 0.001
Visits per month	208.31 (77.64)	231.03 (81.02)	179.90 (63.25)	− 51.13 (22.1)	< 0.001
Completed clinical hours per month	93.04 (24.15)	96.64(23.61)	88.54 (24.31)	− 8.10 (8.4)	0.06
Minutes per visit	28.61 (6.44)	26.82 (6.96)	30.85 (4.94)	4.03 (15.0)	< 0.001
Proportion of available appointments filled	0.79 (0.14)	0.77 (0.14)	0.81 (0.12)	0.04 (5.2)	0.13
EHR use metrics					
Time per completed clinical hour					
Total EHR time	46.94 (16.28)	43.06 (13.79)	51.78 (17.93)	8.72 (20.3)	0.001
Work outside of scheduled clinical hours	8.33 (7.69)	7.00 (6.94)	9.97 (8.30)	2.97 (42.4)	0.01
Time on documentation	13.92 (7.20)	11.82 (5.82)	16.54 (7.94)	4.72 (39.9)	< 0.001
Time on inbox	7.27 (3.55)	6.84 (3.19)	7.80 (3.92)	0.96 (14.0)	0.16
Time on orders	7.92 (2.89)	7.79 (3.38)	8.09 (2.15)	0.30 (3.9)	0.16
Time on chart review	7.78 (3.83)	7.08 (3.25)	8.64 (4.33)	1.56 (22.0)	0.06
Proportion of orders with team contribution	0.17 (0.14)	0.18 (0.16)	0.15 (0.10)	− 0.03 (16.7)	0.88
Proportion of note characters typed or dictated by physician	0.09 (0.07)	0.07 (0.07)	0.11 (0.08)	0.04 (36.4)	0.006
Proportion of note characters contributed by physician vs. other team member	0.84 (0.30)	0.82 (0.33)	0.87(0.24)	0.05 (6.1)	0.91

^*^Comparisons made via χ^2^ tests

### Productivity Differences by Physician Gender

**Figure 1:**
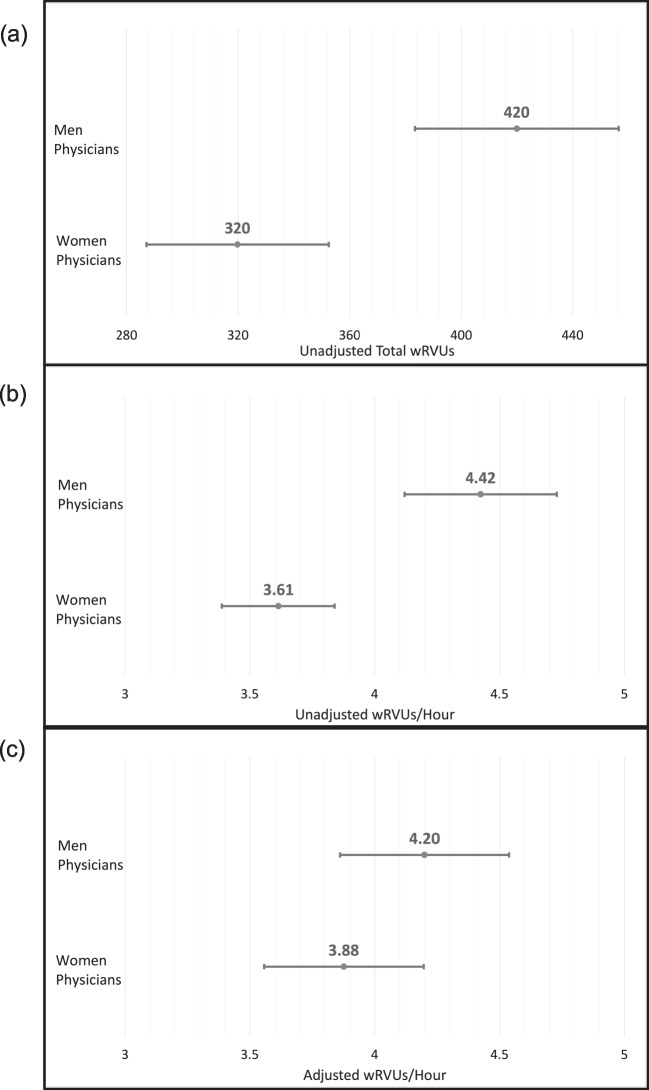
Differences in work RVUs generated by men and women physicians: (**a**) unadjusted wRVUs per month, (**b**) unadjusted wRVUs per hour, and (**c**) adjusted wRVUs per hour. The adjusted model accounts for physician age and gender, panel count, panel gender ratio, panel complexity, proportion of available appointments filled, minutes per visit, EHR work outside of scheduled hours, documentation time, inbox time, time on orders, time on chart review, proportion of orders with team contribution, proportion of note characters typed or dictated by the physician, proportion of note characters with team contribution, COVID wave, and E/M code change

In unadjusted analysis, compared with men physicians, women physicians generated 23.8% fewer monthly wRVUs (319.8 vs. 419.9, *p* < 0.001, Fig. 1a) and 18.0% lower unadjusted wRVUs/hour (3.61 vs. 4.42, *p* < 0.001, Fig. 1b). There were no significant differences in unadjusted wRVUs/visit (1.79 vs. 1.86 wRVUs/visit, *p* = 0.46) between women and men physicians.

### Clinical Activity and EHR Time Differences by Physician Gender

Women physicians had an average of 51 (22.1%) fewer visits per month (*p* < 0.001) and 8 (8.4%) fewer completed clinical hours per month (*p* = 0.06) with 4 min (15.0%) more time spent per visit (*p* = 0.06). In terms of EHR use and team support, women and men physicians spent similar time on inbox, orders, and chart review and had similar team support for order entry and documentation. However, compared with men physicians for every completed clinical hour, women physicians had higher total EHR time (51.78 vs. 43.06 min, *p* = 0.001), work outside of scheduled hours (9.97 vs. 7.00 min, *p* = 0.01), and documentation time (16.54 vs. 11.82 min, *p* < 0.001). Women physicians typed or dictated more note characters than men physicians with a relative difference of 36.4% (11% of characters vs. 7%, *p* = 0.006).

### Factors Associated with wRVU/hour—Multivariable Analyses

In multivariable analysis considering the whole physician population and controlling for physician age, practice characteristics, EHR use, and temporal trends, physician gender was no longer associated with productivity with similar adjusted wRVUs/hour for men and women physicians (4.20 vs. 3.88, IRR 0.92 [95% CI 0.79–1.08], *p* = 0.31, Fig. 1c, Table 2). However, the following variables were associated with higher adjusted wRVUs/hour: a higher proportion of orders with team contribution (IRR 1.41 [1.26–1.58], *p* < 0.001) and a lower proportion of note characters typed or dictated by the physician (IRR 0.53 [0.37–0.77], *p* = 0.001). Also, compared to physicians in the lowest quartile for time on documentation, physicians in the third and fourth quartile had lower wRVUs/hour (IRR 0.95 [0.92–0.99], *p* = 0.005 and IRR 0.94 [0.90–0.99], *p* = 0.011, respectively). Additionally, the E/M code change was associated with 4% higher RVUs/hour across all physicians ([1 to 7%], *p* < 0.001).

**Table 2:** Factors Associated with Physician Productivity (wRVUs/hour) in Generalized Estimating Equation Models

	IRR (95% CI)	***p*-**value
Characteristics		
Physician gender: Women (men as reference)	0.92 (0.79–1.08)	0.31
Physician age (25–44 as reference)		
45–54	1.01 (0.94–1.09)	0.73
55–64	1.07 (0.97–1.17)	0.16
65 +	1.06 (0.96–1.17)	0.26
Practice characteristics		
Panel count	1.00 (1.00–1.00)	0.001
Panel gender ratio, proportion female	1.12 (0.72–1.72)	0.62
Panel complexity	1.03 (0.95–1.10)	0.52
Minutes per visit	0.98 (0.97–0.98)	< 0.001
Proportion of available appointments filled	1.41 (1.22–1.63)	< 0.001
EHR use metrics All time-based metrics are per completed clinical hour		
Quartiles for EHR work outside of scheduled hours		
1st (WOW < 3.28 min)	Reference	
2nd (WOW 3.28–6.70 min)	0.99 (0.96–1.02)	0.64
3rd (WOW 6.70–11.41 min)	1.01 (0.97–1.06)	0.54
4th (WOW > 11.41 min)	1.04 (0.98–1.09)	0.17
Quartiles for time on documentation		
1st (note < 8.85 min)	Reference	
2nd (note 8.85–12.51 min)	0.98 (0.95–1.01)	0.14
3rd (note 12.51–16.93 min)	0.95 (0.92–0.99)	0.005
4th (note > 16.93 min)	0.94 (0.90–0.99)	0.011
Quartiles for time on inbox		
1st (IB < 4.71 min)	Reference	
2nd (IB 4.71–6.53 min)	1.01 (0.97–1.05)	0.74
3rd (IB 6.53–8.99 min)	1.00 (0.96–1.04)	0.99
4th (IB > 8.99 min)	1.02 (0.97–1.08)	0.34
Quartiles for time on orders		
1st (orders < 5.74 min)	Reference	
2nd (orders 5.74–7.32 min)	1.01 (0.97–1.05)	0.57
3rd (orders 7.32–9.40 min)	1.01 (0.98–1.05)	0.44
4th (orders > 9.40 min)	1.02 (0.99–1.06)	0.19
Quartiles for time on chart review		
1st (review < 4.96 min)	Reference	
2nd (review 4.96–6.79 min)	1.01 (0.98–1.03)	0.46
3rd (review 6.79–9.62 min)	1.03 (1.00–1.07)	0.05
4th (review > 9.62 min)	1.03 (0.98–1.07)	0.24
Proportion of orders with team contribution	1.41 (1.26–1.58)	< 0.001
Proportion of note characters typed or dictated by MD	0.53 (0.37–0.77)	0.001
Proportion of note characters contributed by MD vs other team member	1.03 (0.98–1.10)	0.17
Temporal trends		
COVID wave (Feb. 2020 and before as reference)		
March–June 2020	0.87 (0.83–0.91)	< 0.001
July–Oct. 2020	1.01 (0.99–1.04)	0.21
Nov. 2020–March 2021	1.11 (1.08–1.14)	< 0.001
April–June 2021	1.07 (1.04–1.11)	< 0.001
E/M code change (before Jan 2021 as reference)	1.04 (1.01–1.07)	0.02

**Abbreviations**: *CI* confidence interval, *COVID,* coronavirus disease of 2019, *EHR* electronic health record, *E/M* evaluation and management, *IRR* incidence rate ratio, *wRVU* work relative value unit, *WOW* work outside of scheduled hours, *IB* inbox

### Effect of the E/M Code Change by Physician Gender

**Figure 2:**
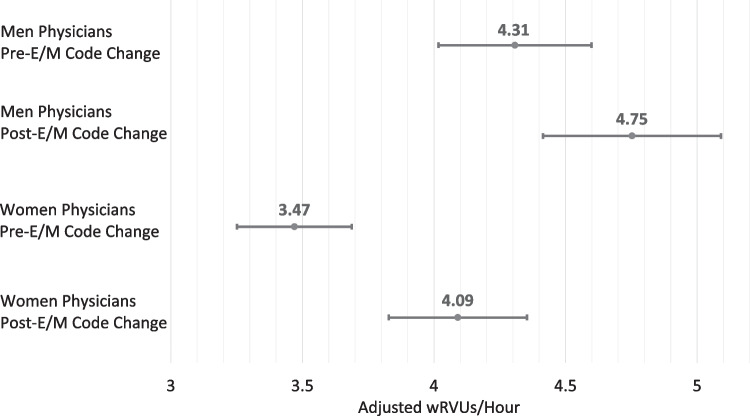
Predictive margins for wRVUs/hour after random effects model including binary variables for gender, 2021 E/M code change, and their interaction

In a secondary analysis to assess whether there was a differential change in productivity by gender associated with the E/M code change, men physicians had 10% higher wRVUs/hour associated with the E/M code change (IRR 1.10 [1.07 to 1.14], *p* < 0.001), and women physicians had an additional 7% greater increase in wRVUs/hour than men physicians (IRR 1.07 [1.02 to 1.12], *p* = 0.009) (marginal effects plotted in Fig. 2). The overall change for women physicians was an increase of 17.9% (13.7 to 22.1%) in wRVUs/hour and, for men, physicians was an increase of 10.3% (6.5 to 14.2%).

## DISCUSSION

### Principal Findings

In this longitudinal study examining monthly physician time and productivity in ambulatory general internal medicine, consistent with prior literature^7, 13–15^, women physicians’ monthly productivity was lower than their male counterparts across fewer visits and more time per visit as well as more EHR time per hour of clinical work. However, after controlling for physician age, practice characteristics, EHR use, and temporal trends, physician gender was no longer associated with hourly physician productivity. Other intervenable EHR factors that were associated with higher hourly physician productivity included (1) a higher proportion of team support on orders, (2) lower proportion of note characters typed or dictated by the physician, (3) less time on documentation, and (4) the 2021 E/M code change. Notably, productivity (wRVUs/hour) increased 10% for men physicians after the E/M code change and 18% for women physicians.

### Findings in Context

Our findings offer insights into factors associated with physician productivity and potential solutions for the physician gender productivity gap in primary care internal medicine. While we found that women physicians completed significantly fewer visits per hour and generated significantly fewer wRVUs/hour as compared to men, after controlling for physician age, practice characteristics, EHR use, and temporal trends, hourly productivity (measured in wRVUs) was similar between men and women physicians. Teamwork on orders, less time on documentation, and shorter notes were associated with increased physician productivity across the whole sample. Interventions that enhance teamwork on orders or documentation, such as use of team-based ordering and scribes, thus represent potentially actionable interventions to enhance productivity of all physicians. Our study also provides an early look at the results of the change in E/M coding. Consistent with previous findings from Apathy et al.,^17^ demonstrating immediate changes in E/M coding patterns after the policy went into effect, we found that the E/M code change was associated with significant increases in clinical productivity per hour across all physicians. We have uniquely demonstrated that the change benefited women physicians more than men physicians. Given evidence that women physicians spend more time listening to patients, providing more preventive care, and counseling,^8^ the change in E/M coding likely enabled physicians to consider time spent on these activities when billing, thus in effect adding more reimbursement for these activities, and potentially explaining the additional benefit of the policy change for women physicians.

Our finding of 14.9% more time per visit being spent by women physicians is consistent with Ganguli et al.’s national cross-sectional analysis^7^ which found a rate of 15.7% more time per visit for women physicians. The finding of an additional hour each day spent on the EHR by women physicians—much of which is spent on documentation and completed outside of scheduled hours—is consistent with multiple other studies’ findings.^12–15^

### Strengths in Relation to Other Studies

This study had several strengths including its gender-balanced sample size of internal medicine physicians on the same EHR, its controlling for temporal trends, and its analysis of the specific EHR activities associated with differences in physician productivity.^7^ It also validates Ganguli et al.’s study on a different EHR vendor (Epic vs. athenahealth) product. With a study period that straddled pre-pandemic through early delta-wave as well as before and after the E/M code change, making conclusions regarding productivity less likely to be confounded by temporal trends.

### Limitations

The study has several limitations. Given the sample size and single practice network, the results may not be generalizable to other specialties, practice settings, or compensation models. Due to data availability, metrics were derived from monthly calculations, limiting visit-level and practice-level fixed effects analyses (limiting ability to compare physicians within the same practice and account for unobserved confounding at the practice level). Therefore, we are unable to determine if women physicians spend more time working in the EHR because they have fewer visits and are comparatively less busy; however, the latter possibility seems less likely since a higher proportion of women’s available appointment slots were filled relative to men (81% vs. 77%) and the bulk of women physicians’ additional EHR time is spent outside of scheduled clinical hours. Although we cannot definitively determine why women’s visits per hour were lower compared to men’s despite having a greater proportion of slots filled, it is likely that women physicians had fewer available appointments and that appointment slots were longer. As in other studies of EHR use measurement, the data used in this analysis is subject to the limitations of vendor-derived data quality.^12, 18^

### Future Work/Policy Implications

Our findings suggest that local practice or health system solutions seeking to increase physician productivity regardless of gender should maximize teamwork on physician EHR tasks. Implementation of workflows that enhance team-based work on the EHR (such as team contributions to orders) and enhanced availability of scribing resources to reduce documentation time may help increase productivity for all clinicians and decrease time spent on the EHR outside of scheduled clinical time.

Our findings also indicate that policy solutions seeking to improve patient outcomes by incentivizing time spent on patient counseling and education may also help to close the physician gender pay gap since women physicians are already doing more of this work—they just have not been historically compensated for it. Indeed, given evidence of better patient outcomes in some contexts^23, 24^, women physicians’ productivity may be better in non-fee-for-service payment models such as value-based contracts. It is additionally critical that policymakers recognize and compensate physicians appropriately for more time intensive activities. Currently, productivity-based compensation models may disincentivize more time intensive activities such as screening and counseling, despite the association of these activities with better outcomes.^23–29^ Our results suggest that the January 2021 E/M code changes make a critical step in this direction by allowing physicians and qualified health professionals to be compensated based on medical decision-making or total time spent on a visit, and this may be particularly beneficial for the productivity of women physicians.

Future work is necessary to (1) validate our findings in other settings; (2) quantify factors associated with the gender productivity gap in other specialties, practice settings, and compensation models; (3) better understand the factors contributing to the fewer patient visits seen by women than men physicians and how time is used differently during visits; and (4) characterize which specific activities women physicians spend more time on than men away from the computer (such as screening and counseling, which may be more time intensive but are associated with enhanced clinical outcomes outcomes).^30, 31^

## CONCLUSIONS

In this study, differences in physician time and productivity by physician gender were driven by patient visit volume, with women physicians having fewer visits per month but spending more time during and after each patient visit. Women physicians spent more time with each patient, using the EHR outside of scheduled hours, and on documentation. Intervenable factors associated with higher productivity were related to increased team support and billing for time-based activities such as patient counseling. In particular, the 2021 E/M code change preferentially benefited women physicians’ hourly productivity. Newer payment models that incentivize longer physician visits and physician–patient communication may benefit patients and secondarily reduce the physician gender pay gap.

## Abbreviations

CI: Confidence interval
COVID: Coronavirus disease of 2019
EHR: Electronic health record
E/M: Evaluation and management
GEEs: Generalized estimating equations
IRR: Incidence rate ratio
PCP: Primary care physician
wRVU: Work relative value unit
